# Identifying genes associated with Sorafenib resistance in hepatocellular carcinoma to develop risk model

**DOI:** 10.1007/s12672-025-03442-x

**Published:** 2025-08-20

**Authors:** Kuan He, Hongyan Hui, Fang Zhou, Kai Liu, Xi Zhao, Jun Liu

**Affiliations:** 1 Department of Surgery, Dongguan Hospital of Guangzhou University of Chinese Medicine, Dongguan, People’s Republic of China; 2 Department of Clinical Pharmacy, The First Affiliated Hospital of Xinxiang Medical University, Weihui, People’s Republic of China; 3 Laboratory Medicine, Dongguan Hospital of Guangzhou University of Chinese Medicine, Songshan Lake Avenue East City Section 3, Dongguan, 523127 People’s Republic of China

**Keywords:** HCC, Sorafenib, Resistance-associated genes, Risk model, Immune cell infiltration

## Abstract

**Background:**

Hepatocellular carcinoma (HCC) poses a considerable global health challenge, notably due to the resistance to sorafenib therapy, which significantly impedes effective treatment strategies. This study aimed to identify potential resistance-associated genes and develop a robust prognostic model to predict patient outcomes.

**Methods:**

We utilized transcriptomic data from the gene expression omnibus (GEO) database, focusing on sorafenib-resistant Huh7 and MHCC97H cell lines (GSE94550 and GSE176151), and integrated expression, mutation, and clinical data from the cancer genome atlas (TCGA) and international cancer genome consortium (ICGC) databases. Single-cell RNA sequencing data (GSE149614) were processed with the Seurat and Harmony R packages for quality control and integration. Differential gene expression analysis, consensus clustering, and principal component analysis were performed to identify significant genes and stratify patients based on prognostic outcomes.

**Results:**

The analysis revealed 305 potential resistance-associated genes, with a seven-gene (ANAPC13, NCAPD2, KIF2C, CDK5RAP2, MANBA, PPAT, and LPCAT1) risk model demonstrating significant prognostic capability, indicated by area under curve values of 0.824, 0.746, and 0.717 for 1, 3, and 5-year survival predictions, respectively. Notably, immune cell infiltration analyses highlighted significant correlations between risk scores and specific immune cell types, suggesting potential therapeutic targets. Drug sensitivity analysis further identified various compounds with lower IC50 values in high-risk groups. To facilitate clinical application, a nomogram plot was designed.

**Conclusion:**

This study provides a comprehensive framework for understanding sorafenib resistance in HCC, alongside a validated prognostic model that holds potential for clinical application.

## Introduction

Hepatocellular carcinoma (HCC) is a major global health issue, ranking sixth in cancer incidence and third in cancer-related deaths [1]. Its strong association with chronic liver diseases, particularly hepatitis B and C, complicates prevention, control, and treatment efforts [2]. Although hepatitis virus vaccines have reduced HCC burden, overall cases continue to rise. Despite remarkable advances in medical science and technology, treating advanced HCC continues to pose difficulties. Despite remarkable advances in medical science and technology, treating advanced HCC continues to pose difficulties. Currently, immunotherapy and targeted therapies are key treatments for advanced HCC, with sorafenib, a multikinase inhibitor, serving as the first-line agent [3]. Sorafenib demonstrates efficacy by promoting apoptosis, inducing ferroptosis, reducing angiogenesis, and inhibiting tumor cell proliferation [4]. However, as clinical practice evolves, sorafenib resistance has emerged as a critical limitation to its effectiveness.

Sorafenib resistance can be categorized into primary and acquired type [5]. Primary resistance means that tumor cells naturally tolerate the drug, reducing its effectiveness. In contrast, acquired resistance develops as tumor cells gradually adapt to the pharmacological environment during treatment, leading to a progressive decline in therapeutic efficacy. This complex form of drug resistance is influenced not only by the biological characteristics of tumor cells but also by interactions within the tumor microenvironment (TME) [6]. Several mechanisms contribute to sorafenib resistance in HCC, such as autophagy, epithelial-mesenchymal transition (EMT), inhibition of ferroptosis, and the survival of tumor-initiating cells [7]. These mechanisms form a complex network that allows tumor cells to escape the cytotoxic effects of sorafenib. For example, in hypoxic conditions, the activation of fibroblasts and hepatic stellate cells can further enhance resistance to sorafenib [8]. The TME is composed of various cell types, such as fibroblasts, immune-inflammatory cells, endothelial cells, and adipocytes, all of which play a role in the development of drug resistance. These cells interact with tumor cells by secreting growth factors, cytokines, and chemokines, which collectively foster the emergence of drug resistance. Additionally, hypoxia not only boosts autophagy, which negatively affects the efficacy of sorafenib, but also promotes resistance through alternative mechanisms [9]. Changes in signaling pathways within tumor cells are crucial in the context of drug resistance. For instance, sorafenib can inhibit the proliferation of HCC cells that are influenced by macrophages by blocking the release of insulin-like growth factor from M2 macrophages [10]. However, tumor cells have the ability to evade the effects of treatment by modifying their internal signaling pathways. Recent research has pinpointed the chemokines CXCL1 and CXCL2 as potential paracrine factors secreted by M2 tumor-associated macrophages, which contribute to sorafenib resistance in HCC cells via the CXCR2/ERK signaling pathway [11]. These discoveries underscore the intricate relationship between resistance-related genes and the TME, both of which play crucial roles in determining patient responses to therapy and their overall prognosis. Consequently, a deeper investigation into drug resistance-related genes and their interactions with the TME is essential for enhancing our understanding of how cancer cells acquire drug resistance. This insight will serve as a scientific foundation for the development of more effective therapeutic strategies, paving the way for innovative methods to predict the prognosis of HCC patients while also aiming to improve their quality of life and survival rates.

In this study, we initiated our research by gathering cell sequencing data related to sorafenib resistance in HCC from the Gene Expression Omnibus (GEO) database, aiming to identify the genes associated with this resistance. Following this, we performed molecular typing using the identified potential resistance genes within the TCGA-LIHC cohort, which enabled us to investigate variations in pathological stratification, tumor gene mutations, prognosis, and relevant pathways across different types. We also created prognostic molecular models based on the resistance-associated genes and assessed their influence on patient outcomes and the TME. The analysis of the risk-prognostic model and gene subtypes provided fresh perspectives on precision treatment strategies for HCC immunotherapy, thereby enhancing clinical decision-making. Moreover, our findings serve as a valuable resource for future investigations into the mechanisms underlying sorafenib resistance and the development of potential therapeutic approaches for HCC.

## Methods and materials

### Data acquisition and processing

In this study, we carefully selected transcriptional data from the GEO database for an in vitro model of sorafenib resistance in the Huh7 HCC cell line (GSE94550) [12]and mRNA profiles comparing MHCC97H control cells to sorafenib-resistant cells (GSE176151) [13]. We downloaded expression data, mutation data, clinical follow-up information, and relevant pathological details for HCC from the TCGA database, alongside additional expression and clinical follow-up data from the International Cancer Genome Consortium (ICGC) database.

### Single-cell analysis

The scRNA-seq data for 21 liver tissue samples from 10 patients with hepatocellular carcinoma (HCC) were downloaded from the Gene Expression Omnibus database (GSE149614: https://www.ncbi.nlm.nih.gov/geo/). These samples were collected from four sites: primary tumor (Tumor, *n* = 10), portal vein tumor thrombus (PVTT, *n* = 2), metastatic lymph node (Lymph, *n* = 1), and non-tumor liver (Normal, *n* = 8) [14]. The scRNA-seq data were processed using the Seurat R package (version 4.4.0). Quality control was conducted to filter out low-quality or dying cells based on the following criteria: nFeature_RNA > 500 and mitochondrial gene expression < 10%. Subsequently, all samples were integrated using the Harmony R package. For principal component analysis, the Seurat FindVariableFeatures function was employed to select the top 2,000 variable genes. Cell clustering was performed using the Seurat FindNeighbors and FindClusters functions, and nonlinear dimensionality reduction was executed using the Seurat RunTSNE function with default settings. The FindAllMarkers function was utilized to identify specific gene markers for each cluster, with parameters set to min.pct = 0.25 and logfc.threshold = 0.25. Finally, cell clusters were annotated using classic markers: T cells (CD3E, CD3D), HSC (ACTA2), hepatocytes (APOE, ALB), endothelial cells (VWF, CLEC4G), B cells (MS4A1, CD79A), plasma cells (MZB1, CD38), macrophages (CD68, MARCO), and monocytes (S100A8, FCN1).

### Identification of drug resistance genes

To identify drug resistance genes, we conducted an in-depth analysis of differential gene expression between resistant and normal cell lines in the GSE94550 and GSE176151 datasets using the limma package. We set the criteria for significant differential genes as |logFC| >1 and p-value < 0.01 to filter potential candidate genes. Following this, we employed Venn diagrams to pinpoint common differential genes present in both datasets, which we then regarded as potential resistance-related genes.

### Enrichment analysis

To gain a clearer understanding of the biological significance of the identified differential genes, we performed Gene Ontology (GO) and Kyoto Encyclopedia of Genes and Genomes (KEGG) pathway analyses with the R package “clusterProfiler,” using a statistical significance threshold of FDR < 0.05. Furthermore, to further explore the gene expression patterns, we conducted Gene Set Variation Analysis (GSVA) using the GSVA package.

### Clustering and PCA analysis

We employed the “ConsensusClusterPlus” R package, a clustering technique based on repetitive sampling for unsupervised cluster analysis, to categorize patients into two subclusters based on the expression matrix of drug resistance-related genes from the TCGA-LIHC cohort. Following this, we conducted principal component analysis (PCA) using the “ggplot2” R package to investigate gene signatures within the HCC subclusters.

### Candidate gene selection and gene characterization

In this study, we utilized a decision tree-based random forest modeling algorithm to identify key genes significantly associated with the prognosis of patients with HCC. The random forest algorithm assesses feature importance by averaging the results from several decision trees. Initially, we identified candidate genes strongly associated with HCC prognosis using univariate Cox regression analysis, applying a stringent criterion of *p* < 0.01. Next, we used the random forest algorithm to rank the importance of these candidate genes. This process allowed us to identify the top 10 as potential key candidates. We then performed undifferentiated combinations of these 10 genes and conducted Kaplan-Meier (K-M) survival analysis for each combination. To assess the statistical significance of these combinations, we applied a negative logarithmic transformation (-log10) to the p-values and ranked the combinations based on these transformed values. This process enabled us to identify statistically significant gene combinations with fewer genes, laying the groundwork for constructing an efficient and accurate survival prediction model.

### Survival analysis

We categorized HCC patients into high-risk and low-risk groups based on the median risk score to compare their prognoses in both the training and validation sets. We conducted a survival analysis using the K-M method. Additionally, we evaluated the predictive accuracy of the risk scores with Receiver Operating Characteristic (ROC) curves, employing the time-dependent ROC approach through the R package “survival-ROC.” Additionally, we employed a Cox proportional hazards regression model to assess the significance of each parameter on overall survival (OS), which refers to the duration of time from diagnosis or start of treatment until death from any cause. To further investigate the relationship between risk scores and prognosis, we performed a two-factor survival analysis combining risk scores with proliferation-related pathways.

### Risk score-based drug discovery

In our study of potential drug candidates for high-risk populations, we used the half-maximal inhibitory concentration (IC50) values for each patient with HCC. These values enabled us to assess therapeutic responses using data from the Genomics of Cancer Drug Sensitivity (GDSC) database. We correlated the risk scores with IC50 values to identify drugs that could benefit high-risk patients.

### Prognostic nomogram construction

The proportional hazards assumption was tested and Cox regression was performed on Riskscore and Stage using the R survival package. Subsequently, a nomogram model was created and visualized with the rms package, followed by calibration analysis. The prognostic model was evaluated using the timeROC package, with visualization carried out via ggplot2. Finally, the prognostic model was fitted with the survival package, and decision curve analysis (DCA) analysis was conducted using the stdca.R package.

### Bioinformatics and statistical analysis

We utilized R software (version 4.4.1, https://www.r-project.org) to analyze data and generate graphs for this study. We assessed survival outcomes using the log-rank test and compared risk scores between the groups with the “wilcox.test” function.

## Results

### Screening for drug resistance genes

**Fig. 1:**
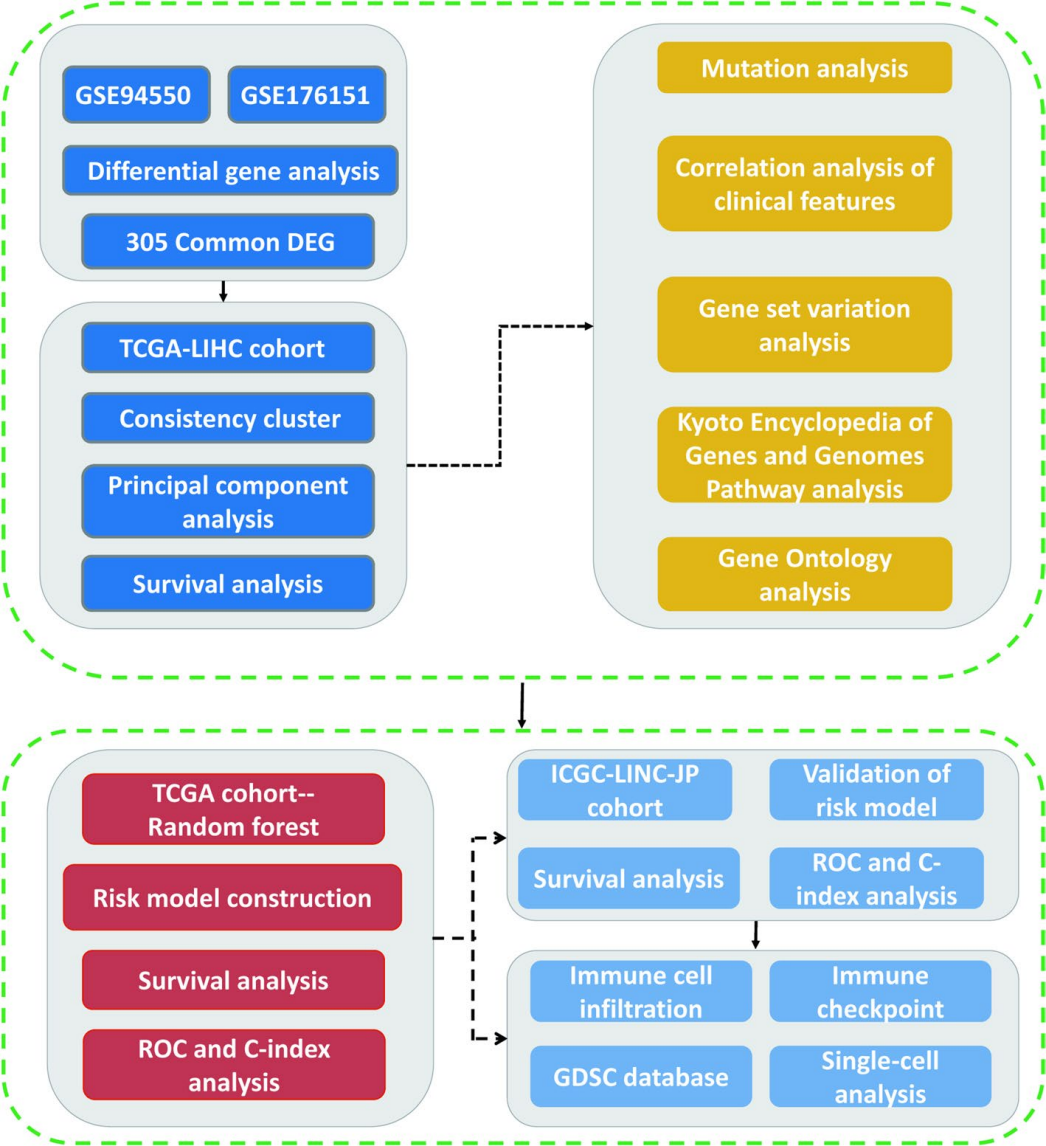
The following flow chart illustrates the design of the study presented in this research

**Fig. 2:**
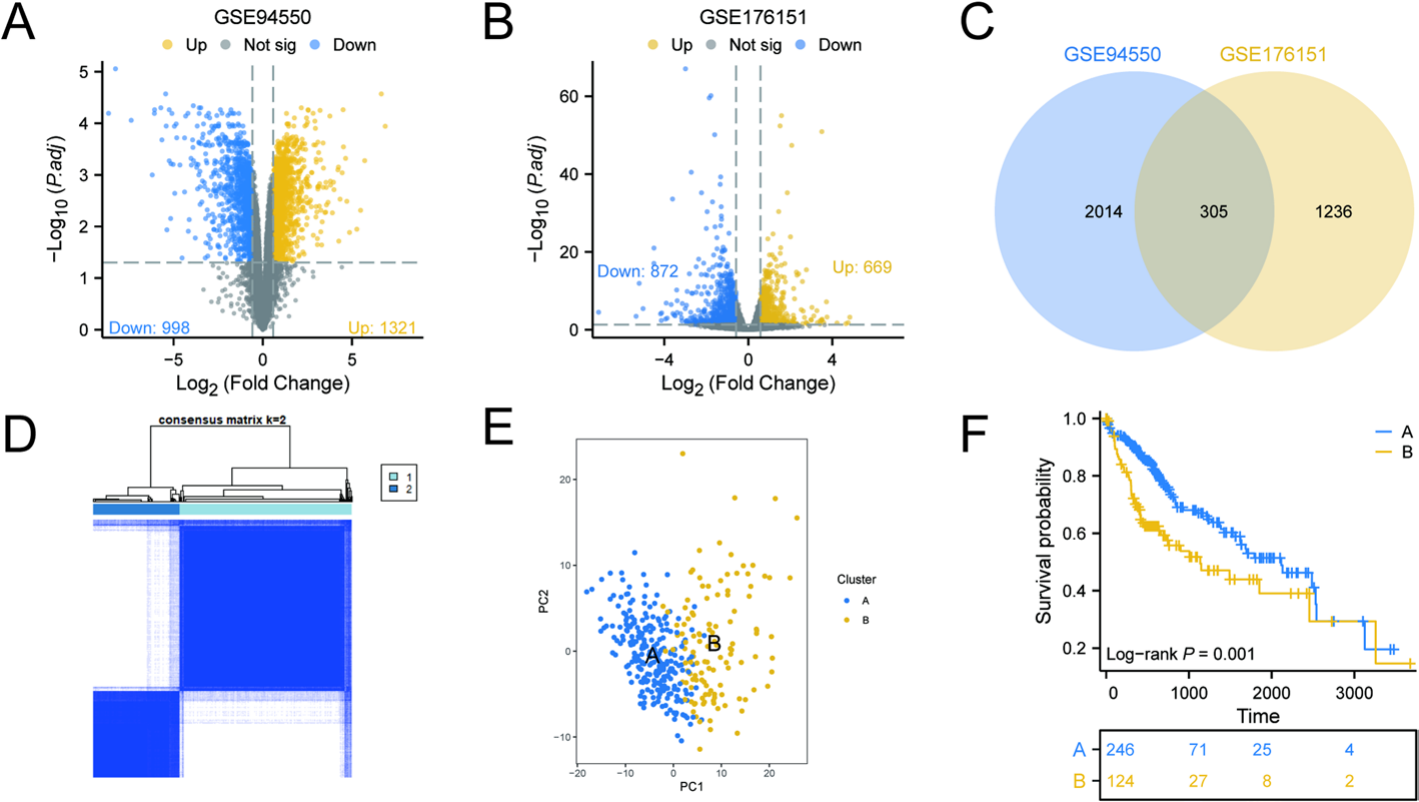
Identification and Cluster Analysis of Genes Related to Resistance to Sorafenib. **A** The volcano plot illustrates the differential genes in Huh7 cells that are either resistant to or sensitive to sorafenib. **B** The volcano plot comparing differential genes between sorafenib-resistant and sorafenib-sensitive MHCC97H cells. **C** The Venn diagram displays the differential genes that are common to both groups of cells. **D** Consensus map of Non-negative Matrix Factorization (NMF) clustering with k = 2 in TCGA-LIHC cohort. **E** PCA was conducted to analyze two distinct clusters within the TCGA-LIHC cohort. **F** Conducting a Kaplan-Meier survival analysis to compare the survival rates between cluster A and cluster B

We obtained transcriptome sequencing data from the GEO database for sorafenib-resistant cell lines, focusing on the Huh7 cell line. This dataset included three resistant lines and three untreated control lines (GSE94450), as well as the GSE176151 dataset, which comprised the MHCC97H cell line with three resistant lines and three control lines. The comprehensive workflow diagram for this study is illustrated in Fig. 1. In the GSE94450 dataset, we identified approximately 998 down-regulated genes and approximately 1,321 up-regulated genes (Fig. 2A). In the GSE176151 dataset, we found approximately 872 down-regulated genes and approximately 669 up-regulated genes (Fig. 2B). By comparing these results with a Venn diagram, we identified approximately 305 differentially expressed genes in both datasets that may be linked to sorafenib resistance in HCC (Fig. 2C). These findings highlight potential targets for further investigation into the mechanisms of drug resistance in HCC.

### Consensus cluster analysis

**Fig. 3:**
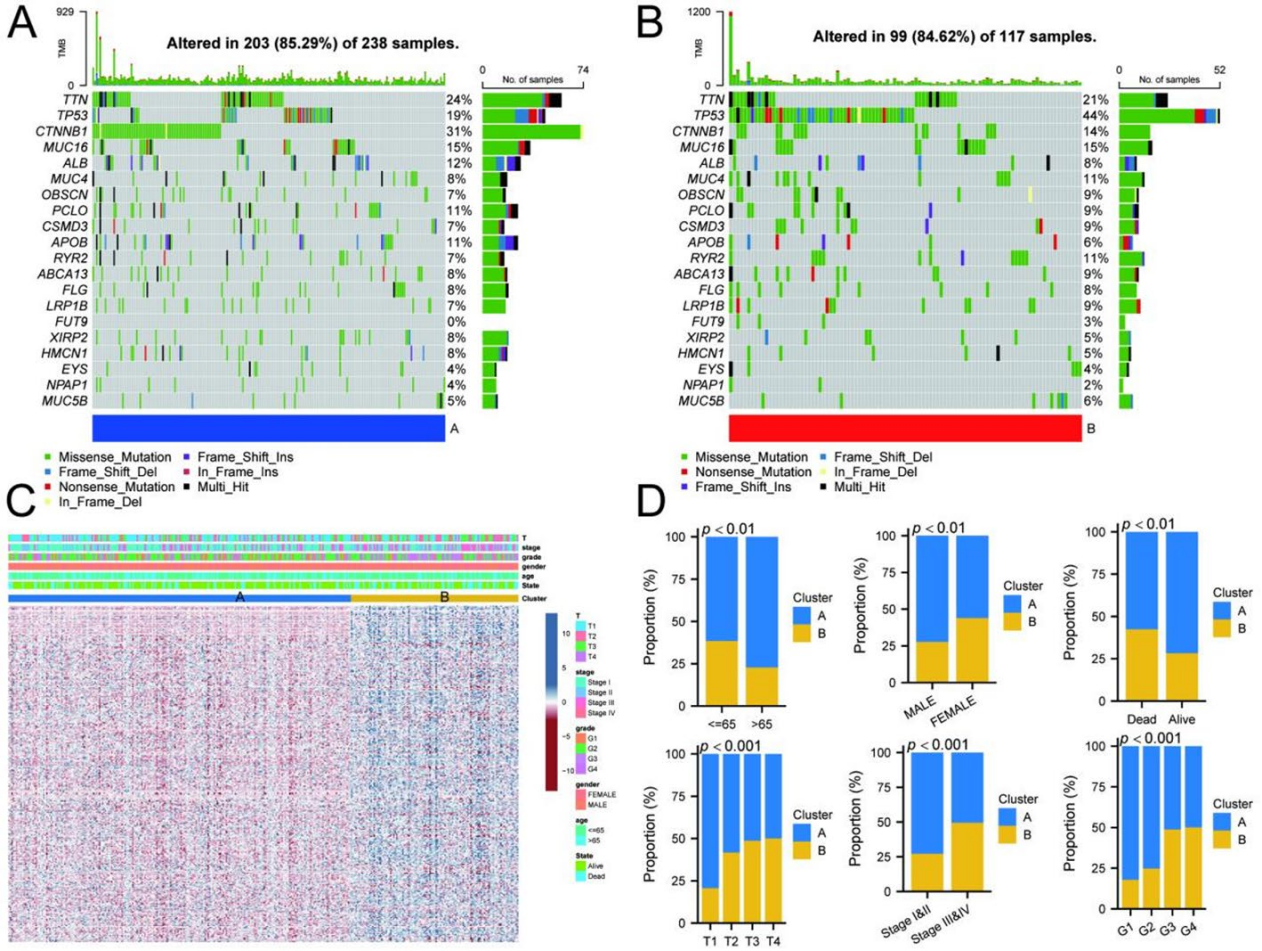
Correlation analysis of mutational landscapes and clinical features in two distinct patient clusters. **A** The mutation landscape of the Cluster A subgroup. **B** The mutation landscape of the Cluster B subgroup. **C** The heatmap provides a detailed correlation analysis between specific clinical features and identified clusters. **D** Distribution of clinical features among different cluster groups

**Fig. 4:**
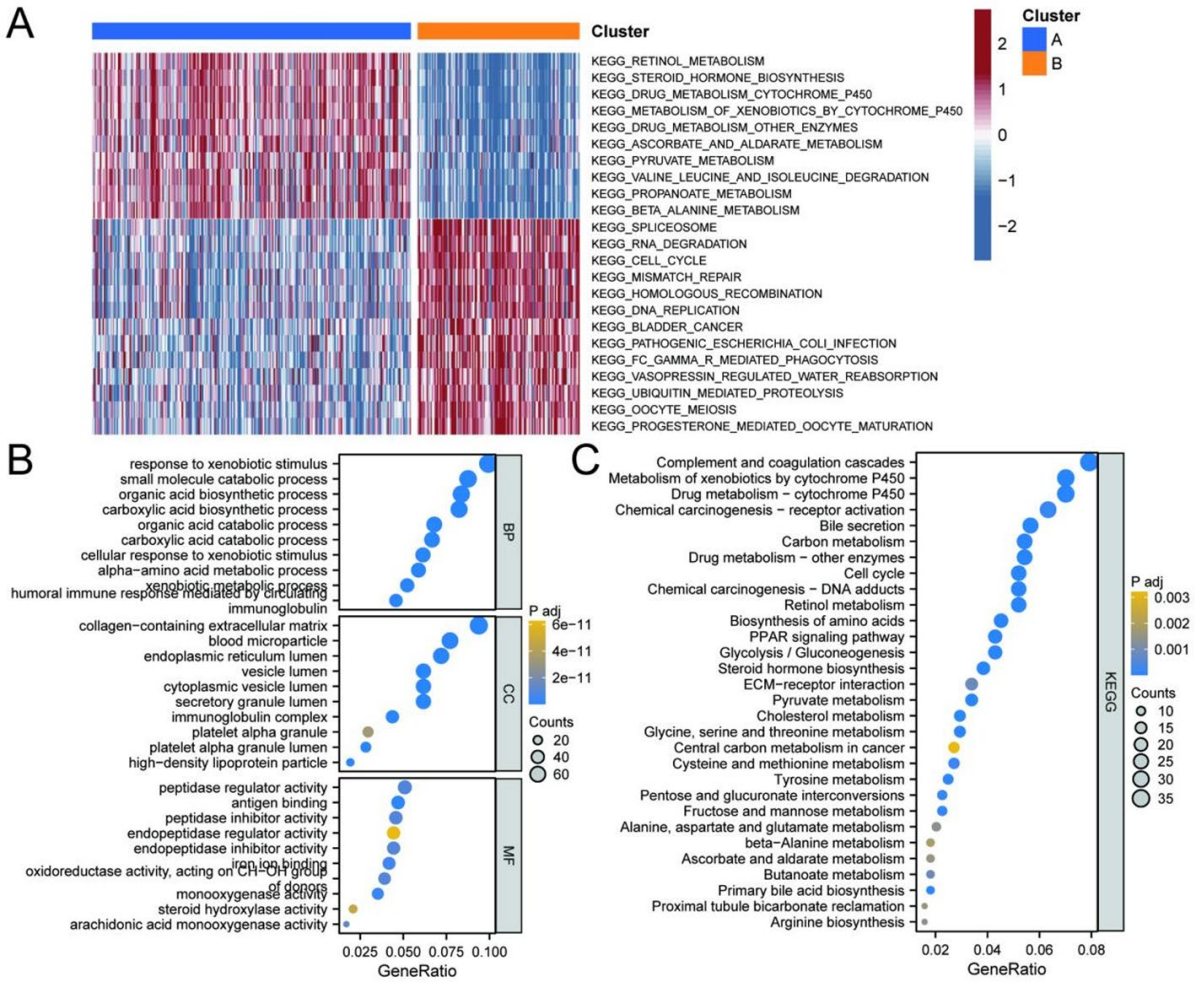
Enrichment analysis of the two clusters. **A** The Gene Set Variation Analysis (GSVA) enrichment analysis was performed in Clusters A and B. **B** GO analysis of differentially expressed genes in two clusters. **C** KEGG analysis of differentially expressed genes in two clusters

To clarify the impact of drug resistance genes on HCC prognosis, we conducted a consensus clustering analysis of 305 potential drug resistance-related genes from the TCGA-LIHC cohort. We tested cluster numbers (K) ranging from 2 to 9. At K = 2, we observed a clear demarcation, indicating that this clustering captured the dataset’s main structural features (Fig. 2D). PCA further confirmed that the two clusters exhibited more distinct differentiation (Fig. 2E). Based on the expression of these 305 genes, we categorized HCC patients into two clusters: Cluster A and Cluster B. K-M survival analysis revealed that the survival rate of Cluster A was significantly better than that of Cluster B (Fig. 2F). Gene mutations play a crucial role in tumorigenesis and prognosis. To gain insight into the survival differences between the two clusters, we analyzed their gene mutations. Notably, Cluster A exhibited a higher mutation rate of CTNNB1, while its TP53 mutation rate was significantly lower compared to Cluster B (Fig. 3A, B). Clinical correlation data further demonstrated significantly different pathological characteristics between the clusters, with Cluster B showing higher mortality rates and more advanced pathological stages (Fig. 3C, D). To explore the potential functional differences between the two subgroups, we conducted GSVA. The major enriched pathways in Cluster A included retinol metabolism, steroid hormone biosynthesis, and pyruvate metabolism. In contrast, Cluster B was primarily enriched in pathways related to spliceosome, RNA degradation, cell cycle, and DNA replication (Fig. 4A). Subsequently, we identified differential genes between the two clusters and analyzed them through GO and KEGG enrichment analyses. The results indicated that these differential genes were involved in small molecule catabolic processes and organic acid biosynthetic processes. The major enriched pathways included drug metabolism, cell cycle, retinol metabolism, and the PPAR signaling pathway (Fig. 4B, C).

### Construction and validation of risk models

**Fig. 5:**
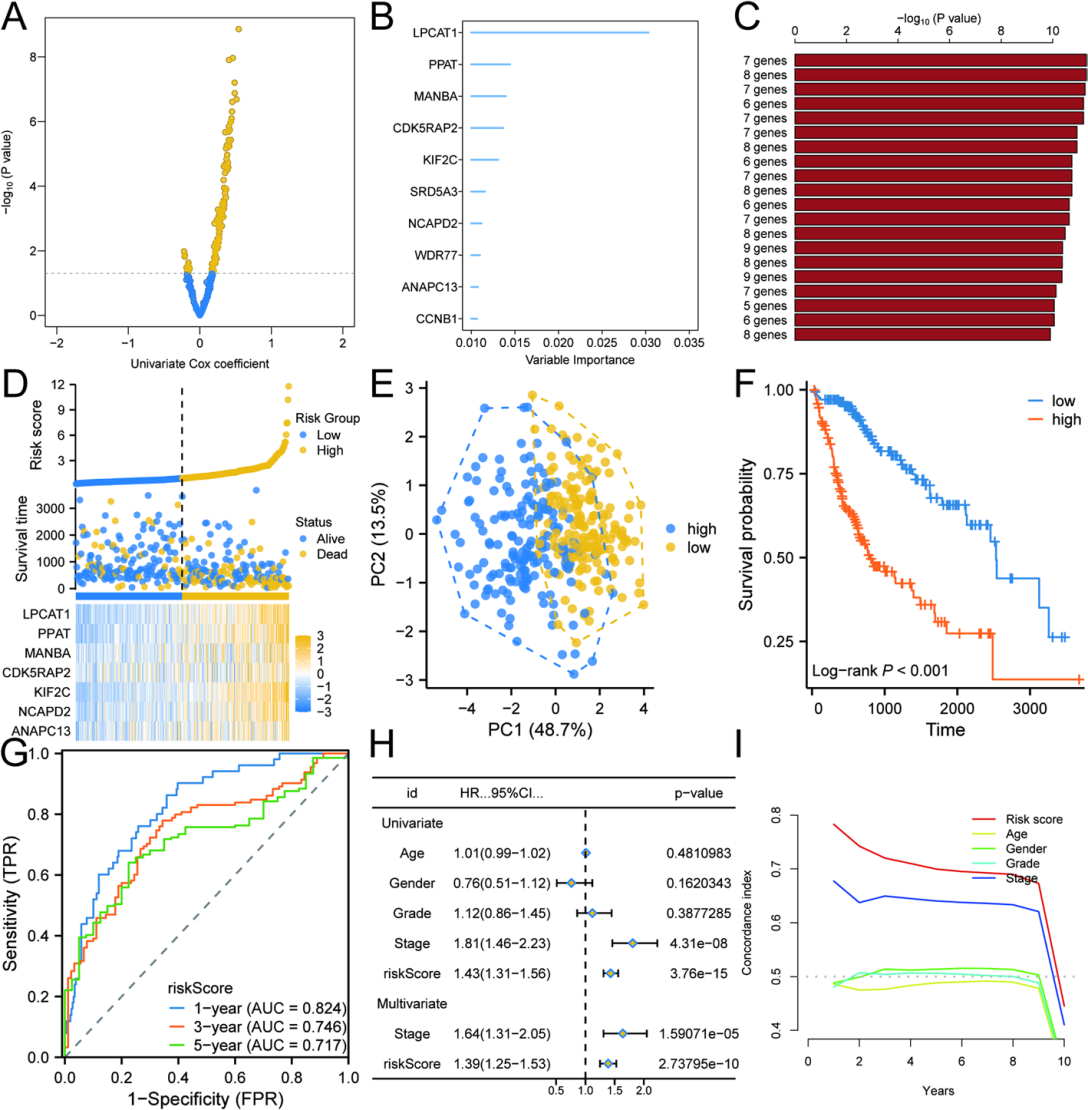
Development and evaluation of prognostic risk models in the TCGA-LIHC cohort. **A** Genes associated with HCC prognosis were identified through univeriate Cox regression analysis of sorafenib resistance-related genes. **B** Random Forest identifies the ten key genes important for prognosis. **C** Prognostic models were developed for various gene combinations and ranked by the significance of the p-values, showcasing the top 20 combinations. **D** The Distribution of Risk Scores, Survival Status, and Gene Expression is Analyzed Between High-Risk and Low-Risk Groups in the TCGA-LIHC Cohort. **E** The PCA analysis clearly showed that the high-risk group differed significantly from the low-risk group. **F** Survival analysis between high and low risk groups. **G** ROC analysis of risk-prognostic models in the TCGA-LIHC cohort. **H** Univariate and multivariate Cox regression analyses of prognostic risk score. **I** Consistency index (C-index) for prognostic risk modeling and clinical characteristics

We screened 128 genes associated with HCC prognosis through univariate Cox regression analysis based on the expression profiles of 305 potential drug resistance genes in the TCGA-LIHC cohort, along with relevant clinical information (Fig. 5A). Next, we assessed the importance of these 128 genes for prognosis using the random forest algorithm, identifying the top 10 prognostic genes: LPCAT1, PPAT, MANBA, CDK5RAP2, KIF2C, SRD5A3, NCAPD2, WDR77, ANAPC13, and CCNB1 ( Fig. 5B). We randomized combinations of these 10 genes to construct prognostic models. The combination yielding the most significant p-value resulted in a 7-gene risk model comprising ANAPC13, NCAPD2, KIF2C, CDK5RAP2, MANBA, PPAT, and LPCAT1 (Fig. 5C). Based on the median risk score from this model, we categorized the cohort into low-risk and high-risk groups. The high-risk group exhibited elevated risk scores and a higher number of deaths (Fig. 5D). PCA demonstrated significant differentiation between the high- and low-risk groups (Fig. 5E). K-M survival analysis revealed a notable difference in survival outcomes, with the high-risk group showing significantly poorer survival rates compared to the low-risk group (Fig. 5F). Subsequently, ROC analysis showed that the area under the curve (AUC) values for this risk model in predicting the 1-, 3-, and 5-year survival of HCC patients were 0.824, 0.746, and 0.717, respectively (Fig. 5G). Univariate and multivariate Cox regression analyses revealed that this risk model is an independent prognostic factor for HCC, irrespective of other clinical variables (Fig. 5H). Additionally, consistency scores demonstrated that this risk model surpassed other clinical factors in predicting survival among HCC patients (Fig. 5I). These findings emphasize the reliability of our risk model in prognostic assessments for HCC, showcasing its potential application in clinical practice.

**Fig. 6:**
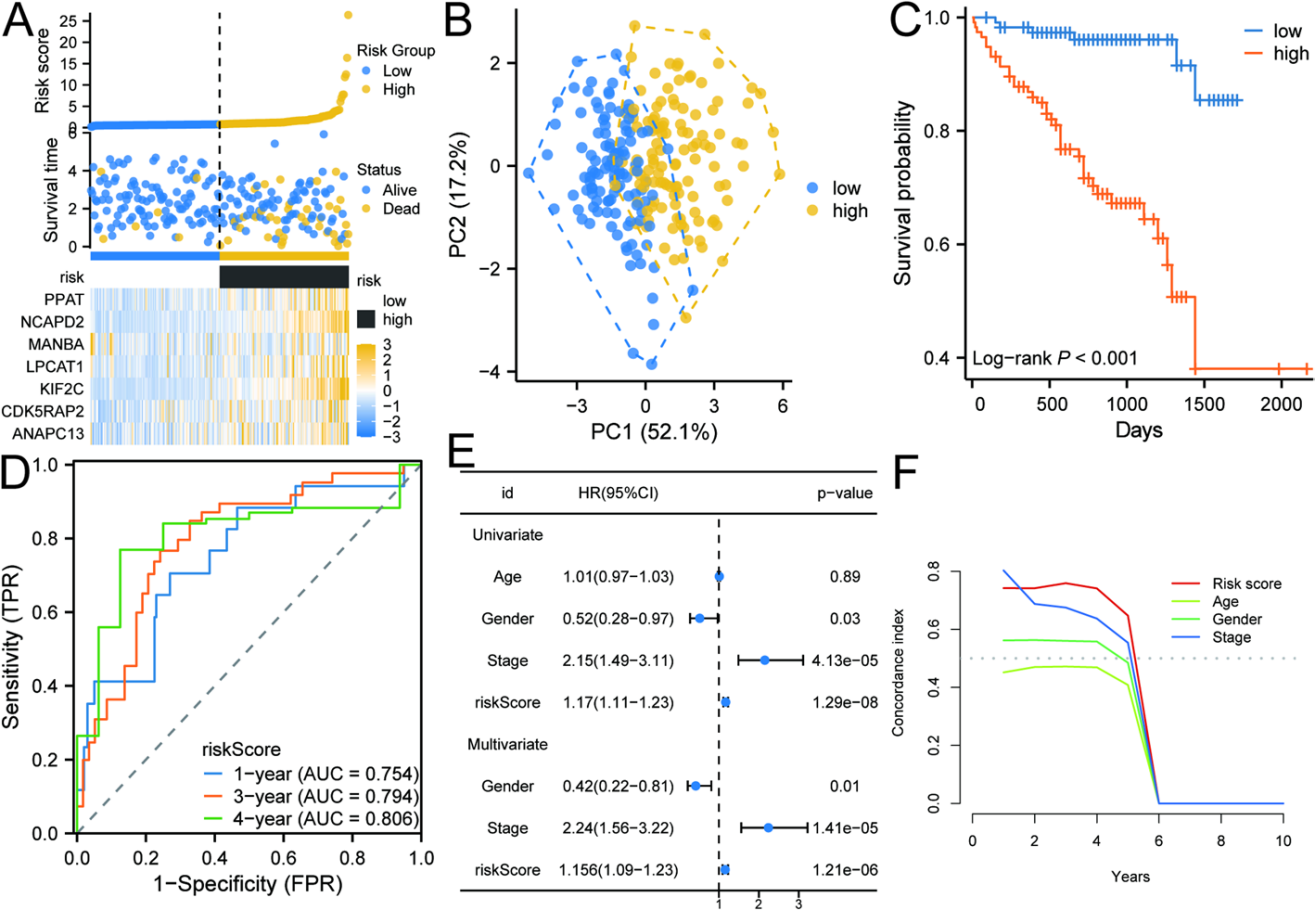
Validation of risk models in the ICGC-LINC-JP cohort. **A** The Distribution of Risk Scores, Survival Status, and Gene Expression is Analyzed Between High-Risk and Low-Risk Groups in the ICGC-LINC-JP Cohort. **B** The PCA analysis revealed significant differences between the high-risk and low-risk groups. **C** A survival analysis comparing the outcomes of high-risk and low-risk groups was conducted. **D** ROC analysis of risk-prognostic models in the ICGC-LINC-JP cohort. **E** Univariate and multivariate Cox regression analyses of prognostic risk score. **F** Consistency index (C-index) for prognostic risk modeling and clinical characteristics

We validated the risk model in the ICGC-LINC-JP cohort, and the results were consistent with those from the TCGA-LIHC cohort. Consistently, the high-risk group showed higher risk scores and more deaths (Fig. 6A). PCA indicated a clear boundary between the high- and low-risk groups, confirming distinct separation (Fig. 6B). K-M survival analysis revealed a significant difference in survival outcomes between the two groups, highlighting the prognostic value of the risk model (Fig. 6C). ROC analysis confirmed the model’s predictive capability, showing AUC values of 0.754, 0.794, and 0.806 for HCC patients at 1, 3, and 4 years, respectively (Fig. 6D). Both univariate and multivariate Cox regression analyses confirmed that the risk model serves as an independent predictor of poor prognosis in HCC patients (Fig. 6E). Furthermore, consistency evaluation demonstrated that the model effectively predicts patient prognosis and outperforms other clinical factors (Fig. 6F). These findings reinforce the reliability and applicability of our risk model in diverse cohorts, underscoring its potential utility in guiding clinical decision-making for HCC patients.

### Immune cell infiltration correlation analysis

**Fig. 7:**
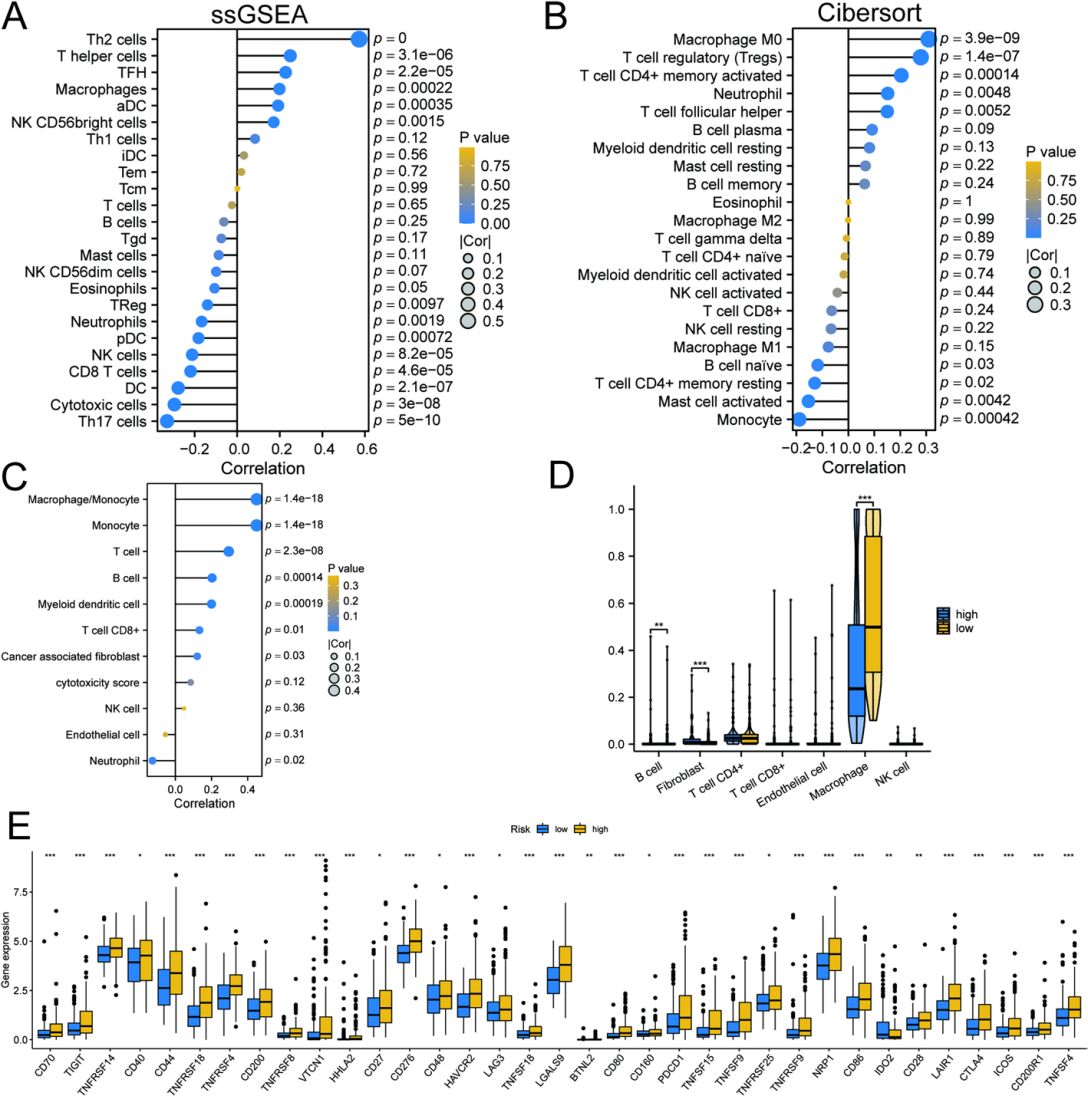
Correlation analysis between immune cell infiltration and risk score. **A** Immune cells were calculated using the ssGSEA algorithm and evaluated for correlation with risk scores. **B** Immune cells were calculated using the Cibersort algorithm and evaluated for correlation with risk scores. **C** Immune cells were calculated using the MCPcounter algorithm and evaluated for correlation with risk scores. **D** Immune cells were calculated using the EPIC algorithm and evaluated for correlation with risk scores. **E** A comparative analysis of immune-related gene expression between high-risk and low-risk groups. *p < 0.05, **p < 0.001, **p < 0.0001

As the importance of immune cells in tumor therapy becomes clearer, we decided to analyze their infiltration status in both high-risk and low-risk groups. In the TCGA-LIHC cohort, we calculated the immune cell scores for each sample using single-sample Gene Set Enrichment Analysis (ssGSEA) and correlated these scores with the risk scores. Our analysis revealed that Th2 cells exhibited a significant positive correlation with the risk score (Fig. 7A), indicating that HCC patients with higher risk scores had greater infiltration of Th2 cells. Additionally, using the CIBERSORT algorithm, we found a significant positive correlation between the risk score and both M0 macrophages and regulatory T cells (Fig. 7B). The MCPcounter algorithm further confirmed a significant positive correlation between the risk score and the presence of macrophages and monocytes (Fig. 7C). According to the EPIC algorithm, the high-risk group had an increased number of fibroblasts, while the counts of CD4 + T cells and macrophages were lower (Fig. 7D). We also investigated the expression levels of immunosuppression-related genes between the high- and low-risk groups. Our findings showed that the expression levels of CD276, CD27, and CTLA4 were significantly higher in the high-risk group compared to the low-risk group (Fig. 7E). These results show a complex relationship between immune cell infiltration and risk stratification in HCC, suggesting potential strategies for targeted immunotherapy in high-risk patients.

### Prediction of potential therapeutic agents

**Fig. 8:**
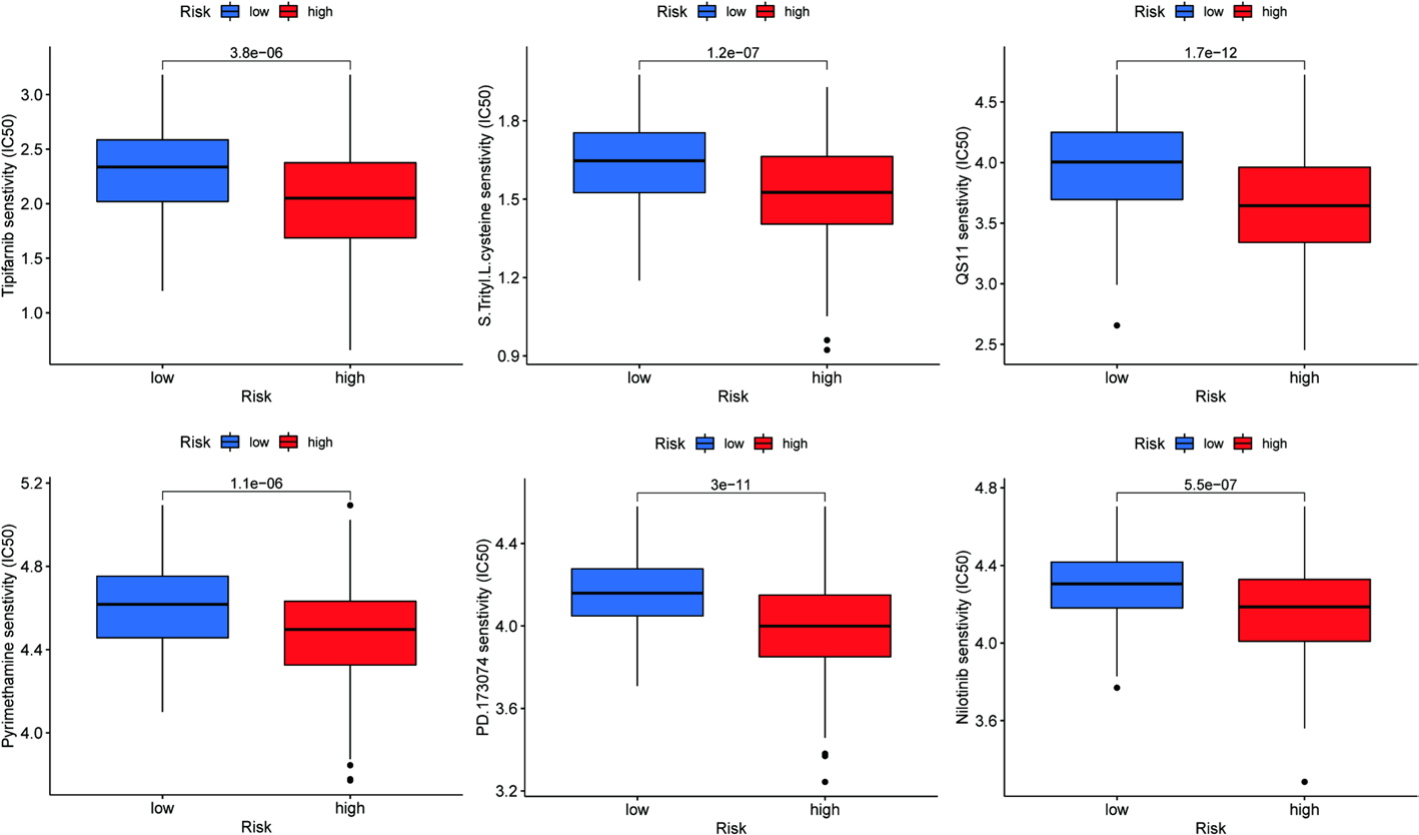
Evaluation of IC50 Values for Different Drugs in High-Risk and Low-Risk Groups Based on the GDSC Database. *p < 0.05, **p < 0.001, **p < 0.0001

The GDSC database provides data on the sensitivity of over 1000 cancer cell lines to 621 anticancer compounds. Our study focused on analyzing patients in the high-risk group who may be resistant to sorafenib. Therefore, we analyzed the sensitivity of different drugs in both the high-risk and low-risk groups. The results showed that Tipifarnib, S-Trityl-L-cysteine, QS11, Pyrimethamine, PD-173,074, and Nilotinib exhibited lower IC50 values in the high-risk group (Fig. 8).

### Construction and evaluation of a nomogram

**Fig. 9:**
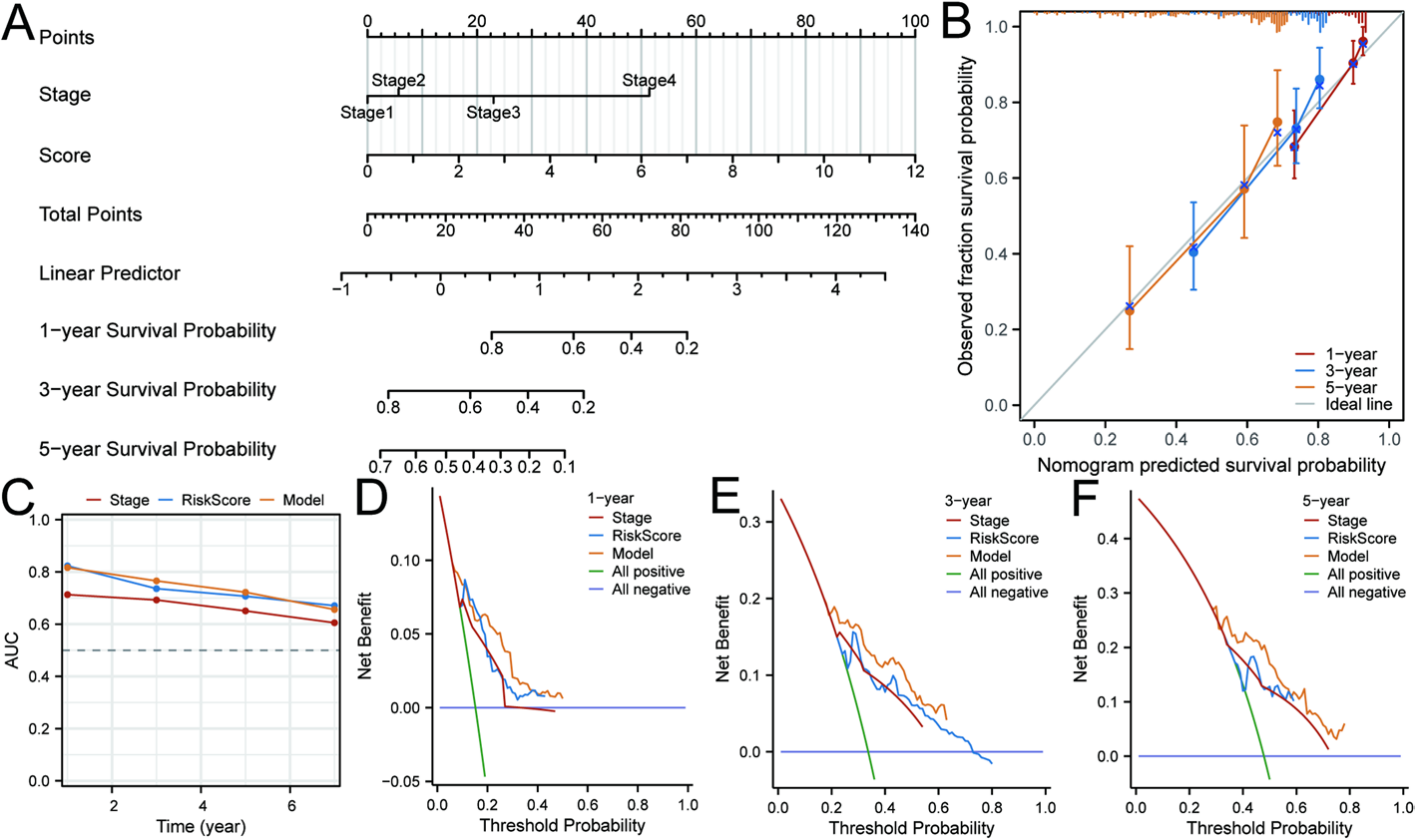
Construction of the nomogram prognostic model. **A** Risk scores and stage were combined to construct the nomogram model. **B** The nomogram calibration curve for 1, 3, 5-year survival time. **C** The risk score, stage, and the model’s area under the curve (AUC). The results of the decision curve analysis, the nomogram, the risk score, and the Stage were presented at 1 (**D**), 3 (**E**), and 5 (**F**) years

In clinical practice, accurately predicting the overall survival of HCC patients is essential for personalized treatment and patient management. We have developed a practical prediction method: a nomogram that integrates risk scores and pathological stages (Fig. 9A). This tool offers clinicians a quantitative way to assess HCC prognosis. We validated its predictive accuracy using calibration curves, which show the model’s consistency with actual outcomes. The nomogram’s predicted survival probabilities closely matched the observed outcomes for 1-year, 3-year, and 5-year survival in HCC patients, demonstrating its reliability and clinical utility (Fig. 9B). Utilizing ROC analysis, our study determined that the integrated nomogram exhibited enhanced predictive performance for the 2- to 5-year overall survival rates of HCC patients, surpassing that of individual risk scores and clinical staging (Fig. 9C). Additionally, DCA substantiated the clinical utility of the combined nomogram, demonstrating that it provides greater net benefits in the prediction process compared to the use of clinical staging or risk scores in isolation (Fig. 9D–F).

### Single-cell analysis

**Fig. 10:**
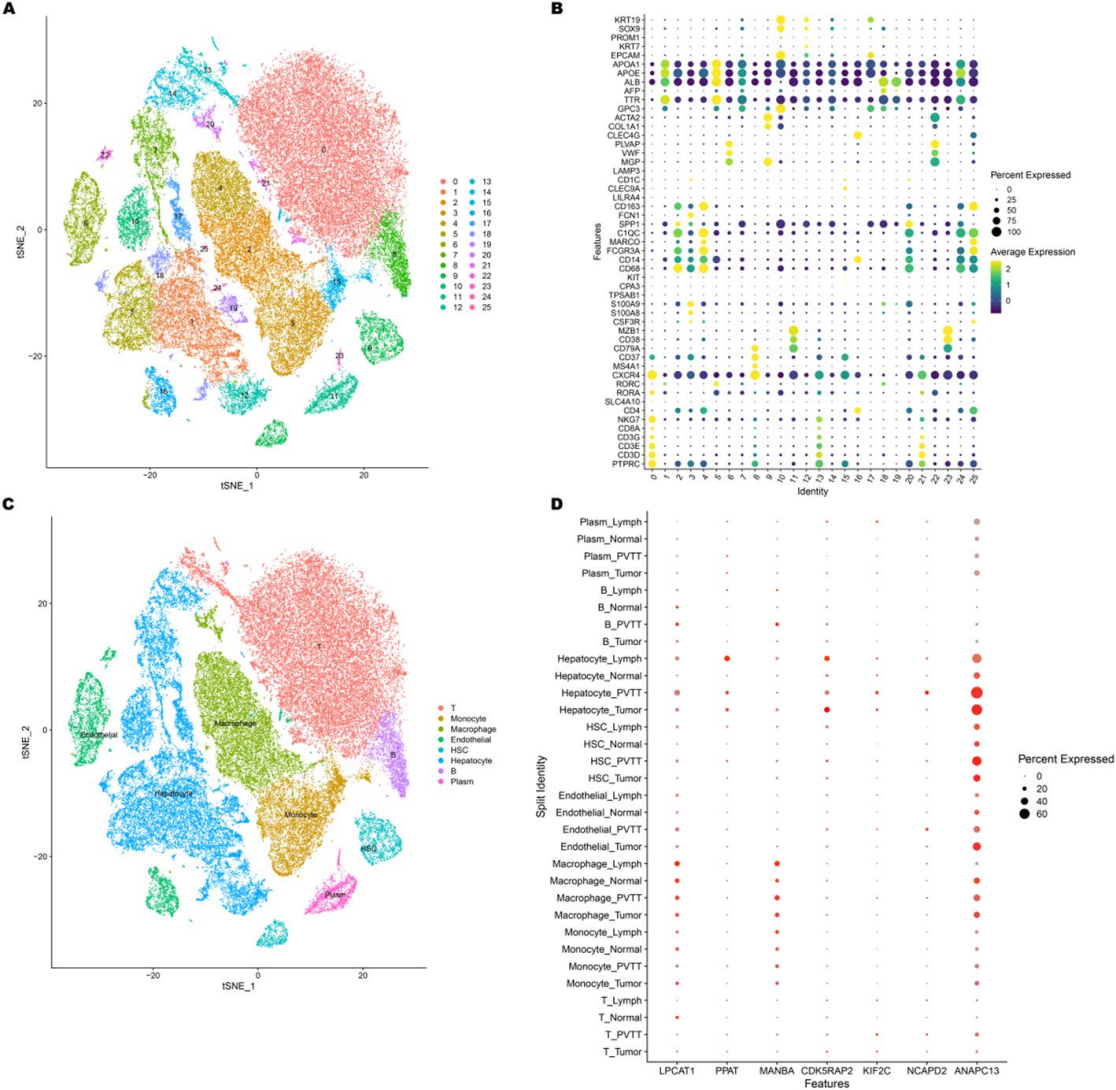
Single-cell analysis of prognostic genes. **A** The tSNE plot shows 25 separate cell clusters. **B** The bubble map demonstrates the expression patterns of marker genes in each cell cluster. **C** Cells were classified into eight distinct categories. **D** The distribution and expression levels of seven prognostic model genes in eight cell types

After quality control, 67,060 cells were used for further analysis. These cells were categorized into 26 clusters (Fig. 10A). Based on the transcript levels of typical marker genes, the cells were classified into eight major types: 23,532 T cells (CD3E, CD3D), 2033 hematopoietic stem cells (ACTA2), 18,603 hepatocytes (APOE, ALB), 3742 endothelial cells (VWF, CLEC4G), 2174 B cells (MS4A1, CD79A), 1655 plasma cells (MZB1, CD38), 9484 macrophages (CD68, MARCO), and 5837 monocytes (S100A8, FCN1) (Fig. 10B, C). The expression of LPCAT1, PPAT, MANBA, CDK5RAP2, KIF2C, NCAPD2, and ANAPC13 is shown. LPCAT1 exhibited relatively high expression in both macrophages and hepatocytes. PPAT showed elevated expression predominantly in hepatocytes. Similarly, MANBA had a higher expression level in macrophages. CDK5RAP2, KIF2C, and NCAPD2 were all found to have relatively high expression in hepatocytes. Finally, ANAPC13 displayed increased expression in both hepatocytes and endothelial cells (Fig. 10D).

## Discussion

Sorafenib is the first targeted drug approved for treating advanced HCC [15]. It works by inhibiting several pathways that contribute to tumor growth and blood vessel formation. Clinical trials show that sorafenib significantly prolongs survival in patients with advanced HCC [16]. Despite its proven effectiveness, the development of drug resistance limits its overall impact. The mechanisms behind sorafenib resistance in HCC are not fully understood. Additionally, there are no effective predictors of treatment response available.

This study conducted a thorough analysis of transcriptomic data from various cell lines, resulting in the identification of 305 potential genes associated with resistance to sorafenib. We categorized HCC patients into two distinct groups, referred to as Cluster A and Cluster B, based on the expression levels of these genes within the TCGA-LIHC dataset. Our findings revealed significant disparities in survival outcomes between the two clusters, with Cluster B exhibiting a poorer prognosis and a higher incidence of TP53 mutations. This finding aligns with earlier research indicating a positive correlation between TP53 mutations and resistance to sorafenib [17]. Additionally, pathway enrichment analysis highlighted that pathways associated with drug and small molecule metabolism were notably enriched in both clusters. These outcomes imply that the 305 differentially expressed genes could be pivotal in the emergence of sorafenib resistance in hepatocellular carcinoma HCC. To explore the potential mechanisms of sorafenib resistance and identify predictive markers of efficacy, we employed Cox regression, random forest algorithms, and other methodologies, ultimately identifying a prognostic model consisting of seven genes. The model showed strong discrimination and validity, with an accuracy of 0.824 in predicting the 1-year survival of HCC patients, making it an independent predictor of poor prognosis. We further validated the stability and effectiveness of the model in an independent external cohort, ICGC-LINC-JP. The model achieved AUC values of 0.754, 0.794, and 0.806 for predicting 1-, 3-, and 4-year survival of HCC patients, respectively, demonstrating robust predictive capability. Zhang S et al. developed a 7-gene risk model based on mitochondrial UPR-related genes for predicting drug resistance, while our study focused specifically on genes linked to sorafenib resistance, offering a more targeted approach. Our model outperformed Zhang S et al.‘s in prediction efficiency, suggesting better identification of patients at risk of sorafenib resistance [18]. Luo T et al. used a broader set of 19 sorafenib resistance-related genes, which, although comprehensive, may complicate clinical application. In contrast, our streamlined gene set maintains high predictive accuracy and is more practical for clinical use [19]. These findings suggest that our prognostic model, built on genes related to sorafenib resistance, possesses strong predictive power.

Studies have shown that the TME, cancer stem cells, and EMT significantly interact, affecting HCC cells’ tolerance to sorafenib [20, 21]. In the HCC microenvironment, the infiltration and types of T cells play a critical role in immune escape and therapeutic resistance [22]. Numerous studies indicate that in various solid tumors, including HCC, infiltrating T cells mainly consist of tumor-promoting TH2 cells and regulatory T cells [23, 24]. These cells play a crucial role in supporting tumor growth by releasing pro-tumorigenic cytokines. In particular, TH2 cells foster an immunosuppressive environment within tumors by producing cytokines such as IL-4 and IL-5 [25]. These cytokines not only promote the survival of HCC cells but also enhance their resistance to drugs. As a result, the presence of TH2 cells may diminish the effectiveness of other anti-tumor T cells, including cytotoxic T cells and TH1 cells. Our research has uncovered a strong positive correlation between the risk score and the infiltration of TH2 cells, indicating that these cells play a significant role in the resistance to sorafenib and contribute to a poor prognosis for patients. These findings offer valuable insights into the mechanisms underlying therapeutic resistance in HCC and highlight potential strategies for future treatments.

A prognostic model based on seven genes associated with sorafenib resistance—ANAPC13, NCAPD2, KIF2C, CDK5RAP2, MANBA, PPAT, and LPCAT1—offers a promising foundation for personalized treatment of HCC patients and may enhance the accuracy of prognostic assessments. NCAPD2 is crucial for cell division, influencing chromosome stability and microtubule polymerization. Its overexpression was linked to poor prognosis in HCC patients, potentially facilitating tumor development by impacting cell proliferation and apoptosis [26, 27]. Regulation of NCAPD2 may also affect HCC cell responses to sorafenib, especially when cell cycle and apoptotic pathways are altered. KIF2C, a protein associated with microtubule motility, plays a significant role in cytokinesis [28]. High expression levels of KIF2C were commonly associated with tumor malignancy and poor patient outcomes. Inhibition of KIF2C expression significantly reduced the proliferation and migratory capacity of HCC cells [29]. CDK5RAP2 is involved in cell cycle regulation and microtubule organization [30]. CDK5RAP2 has been found to be aberrantly expressed in oral squamous cell carcinoma and colon cancer, and promotes the EMT and metastasis of cancer cells [31, 32]. MANBA is implicated in polysaccharide metabolism, which is vital for cellular signaling and metabolism [33]. PPAT, an enzyme involved in purine metabolism, is essential for cellular energy metabolism [34]. ANAPC13, also referred to as anaphase promoting complex subunit 13, is a crucial cation exchange protein in human cells, playing a significant role in various biological processes [35, 36]. However, the expression and function of CDK5RAP2, MANBA, PPAT and ANAPC13 in HCC have not been reported. LPCAT1 is associated with phospholipid metabolism and plays a role in the construction of cell membranes and signaling [37, 38]. Studies have shown that LPCAT1 is upregulated in HCC cells, potentially enhancing the invasive and migratory capabilities of tumor cells [39, 40]. Changes in LPCAT1 expression may further contribute to the resistance of HCC cells to sorafenib by affecting cell membrane fluidity and signaling. The functions of these seven genes are closely tied to the survival, proliferation, and drug tolerance of HCC cells, although their specific mechanisms in HCC require further investigation. They may be involved in the development of sorafenib resistance through the regulation of cell cycle, metabolism, and signaling pathways. A deeper exploration of these genes could provide insights into drug resistance mechanisms in HCC and identify potential targets for novel therapeutic strategies.

We analyzed the GDSC database and identified potential therapeutic agents, including Tipifarnib and S-Trityl-L-cysteine, which exhibit lower IC50 values in the high-risk group. These findings suggest new treatment directions for hepatocellular carcinoma, warranting further validation of their efficacy and mechanisms in clinical studies. Overall, this study enhances our understanding of drug resistance mechanisms in HCC and offers crucial theoretical guidance for future clinical applications. Additionally, we developed a nomogram by integrating the risk score with clinical staging. This integrated nomogram enhances the accuracy of survival predictions by incorporating multiple predictive factors. DCA further underscores the significant potential of this nomogram in clinical practice.

This study is subject to several limitations. Firstly, although we identified numerous differentially expressed genes associated with sorafenib resistance using bioinformatics approaches, these findings were not corroborated through experimental validation. This shortcoming diminishes the biological relevance and practical applicability of the results. Secondly, the limited sample size may have compromised the statistical robustness and generalizability of the findings. Furthermore, the use of data from multiple public databases may introduce batch effects, potentially affecting the reliability of the results. Consequently, future research should incorporate larger, multicenter clinical samples and laboratory validation to more effectively substantiate the role of these genes in drug resistance in HCC.

In summary, this study identified key genes related to sorafenib resistance and developed an effective prognostic risk model, contributing significantly to the field of HCC treatment.

## Data Availability

Data is provided within the manuscript or supplementary information files.

## References

[1] Toh MR, Wong EYT, Wong SH, Ng AWT, Loo LH, Chow PK, Ngeow J. Global Epidemiology and Genetics of Hepatocellular Carcinoma. Gastroenterology. 2023;164:766–82. 10.1053/j.gastro.2023.01.033. 10.1053/j.gastro.2023.01.033 36738977

[2] Younossi ZM, Wong G, Anstee QM, Henry L. The global burden of liver disease. Clin Gastroenterol Hepatol. 2023;21:1978–91. 10.1016/j.cgh.2023.04.015. 37121527 10.1016/j.cgh.2023.04.015

[3] Tang W, Chen Z, Zhang W, Cheng Y, Zhang B, Wu F, Wang Q, Wang S, Rong D, Reiter FP, De Toni EN, Wang X. The mechanisms of Sorafenib resistance in hepatocellular carcinoma: theoretical basis and therapeutic aspects. Signal Transduct Target Ther. 2020;5:87. 10.1038/s41392-020-0187-x. 32532960 10.1038/s41392-020-0187-x PMC7292831

[4] Huang A, Yang XR, Chung WY, Dennison AR, Zhou J. Targeted therapy for hepatocellular carcinoma. Signal Transduct Target Ther. 2020;5:146. 10.1038/s41392-020-00264-x. 32782275 10.1038/s41392-020-00264-x PMC7419547

[5] Ladd AD, Duarte S, Sahin I, Zarrinpar A. Mechanisms of drug resistance in HCC. Hepatology. 2024;79:926–40. 10.1097/HEP.0000000000000237. 36680397 10.1097/HEP.0000000000000237

[6] Xia S, Pan Y, Liang Y, Xu J, Cai X. The microenvironmental and metabolic aspects of Sorafenib resistance in hepatocellular carcinoma, ebiomedicine. 51 (2020) 102610. 10.1016/j.ebiom.2019.102610 10.1016/j.ebiom.2019.102610 PMC7000339 31918403

[7] Fornari F, Giovannini C, Piscaglia F, Gramantieri L. Elucidating the molecular basis of Sorafenib resistance in HCC: current findings and future directions. J Hepatocell Carcinoma. 2021;8:741–57. 10.2147/JHC.S285726. 34239844 10.2147/JHC.S285726 PMC8260177

[8] Shi Y, Shang J, Li Y, Zhong D, Zhang Z, Yang Q, Lai C, Feng T, Yao Y, Huang X. ITGA5 and ITGB1 contribute to Sorafenib resistance by promoting vasculogenic mimicry formation in hepatocellular carcinoma. Cancer Med. 2023;12:3786–96. 10.1002/cam4.5110. 35946175 10.1002/cam4.5110 PMC9939139

[9] Zhang D, Wu F, Song J, Meng M, Fan X, Lu C, Weng Q, Fang S, Zheng L, Tang B, Yang Y, Tu J, Xu M, Zhao Z, Ji J. A role for the NPM1/PTPN14/YAP axis in mediating hypoxia-induced chemoresistance to Sorafenib in hepatocellular carcinoma. Cancer Cell Int. 2022;22:65. 10.1186/s12935-022-02479-0. 35135548 10.1186/s12935-022-02479-0 PMC8822852

[10] Sprinzl MF, Puschnik A, Schlitter AM, Schad A, Ackermann K, Esposito I, Lang H, Galle PR, Weinmann A, Heikenwalder M, Protzer U. Sorafenib inhibits macrophage-induced growth of hepatoma cells by interference with insulin-like growth factor-1 secretion. J Hepatol. 2015;62:863–70. 10.1016/j.jhep.2014.11.011. 25463538 10.1016/j.jhep.2014.11.011

[11] Wang HC, Haung LY, Wang CJ, Chao YJ, Hou YC, Yen CJ, Shan YS. Tumor-associated macrophages promote resistance of hepatocellular carcinoma cells against Sorafenib by activating CXCR2 signaling. J Biomed Sci. 2022;29:99. 10.1186/s12929-022-00881-4. 36411463 10.1186/s12929-022-00881-4 PMC9677647

[12] Regan-Fendt K, Li D, Reyes R, Yu L, Wani N. A., Hu P., Jacob S. T., Ghoshal K., Payne P. R. O., Motiwala T. Transcriptomics-Based drug repurposing approach identifies novel drugs against Sorafenib-Resistant hepatocellular carcinoma. Cancers (Basel). 2020;12. 10.3390/cancers12102730. 10.3390/cancers12102730 PMC7598246 32977582

[13] Sha Y, Pan M, Chen Y, Qiao L, Zhou H, Liu D, Zhang W, Wang K, Huang L, Tang N, Qiu J, Huang A, Xia J. PLEKHG5 is stabilized by HDAC2-related deacetylation and confers Sorafenib resistance in hepatocellular carcinoma. Cell Death Discov. 2023;9:176. 10.1038/s41420-023-01469-z. 37248230 10.1038/s41420-023-01469-z PMC10227013

[14] Lu Y, Yang A, Quan C, Pan Y, Zhang H, Li Y, Gao C, Lu H, Wang X, Cao P, Chen H, Lu S, Zhou G. A single-cell atlas of the multicellular ecosystem of primary and metastatic hepatocellular carcinoma. Nat Commun. 2022;13:4594. 10.1038/s41467-022-32283-3. 35933472 10.1038/s41467-022-32283-3 PMC9357016

[15] Brunetti O, Gnoni A, Licchetta A, Longo V, Calabrese A, Argentiero A, Delcuratolo S, Solimando AG, Casadei-Gardini A, Silvestris N. Predictive and prognostic factors in HCC patients treated with Sorafenib. Medicina (Kaunas). 2019. 10.3390/medicina55100707. 10.3390/medicina55100707 PMC6843290 31640191

[16] Qin S, Bi F, Gu S, Bai Y, Chen Z, Wang Z, Ying J, Lu Y, Meng Z, Pan H, Yang P, Zhang H, Chen X, Xu A, Cui C, Zhu B, Wu J, Xin X, Wang J, Shan J, Chen J, Zheng Z, Xu L, Wen X, You Z, Ren Z, Liu X, Qiu M, Wu L, Chen F. Donafenib versus Sorafenib in First-Line treatment of unresectable or metastatic hepatocellular carcinoma: A randomized, Open-Label, Parallel-Controlled phase II-III trial. J Clin Oncol. 2021;39:3002–11. 10.1200/JCO.21.00163. 34185551 10.1200/JCO.21.00163 PMC8445562

[17] Kam CS, Ho DW, Ming VS, Tian L, Sze KM, Zhang VX, Tsui YM, Husain A, Lee JM, Wong CC, Chan AC, Cheung TT, Chan LK, Ng IO. PFKFB4 drives the oncogenicity in TP53-Mutated hepatocellular carcinoma in a Phosphatase-Dependent manner. Cell Mol Gastroenterol Hepatol. 2023;15:1325–50. 10.1016/j.jcmgh.2023.02.004. 36806581 10.1016/j.jcmgh.2023.02.004 PMC10140800

[18] Zhang S, Guo H, Wang H, Liu X, Wang M, Liu X, Fan Y, Tan K. A novel mitochondrial unfolded protein response-related risk signature to predict prognosis, immunotherapy and Sorafenib sensitivity in hepatocellular carcinoma. Apoptosis. 2024;29:768–84. 10.1007/s10495-024-01945-6. 38493408 10.1007/s10495-024-01945-6

[19] Luo T, Chen X, Pan W, Zhang S, Huang J. The Sorafenib resistance-related gene signature predicts prognosis and indicates immune activity in hepatocellular carcinoma. Cell Cycle. 2024;23:150–68. 10.1080/15384101.2024.2309020. 38444181 10.1080/15384101.2024.2309020 PMC11037289

[20] Xia P, Zhang H, Xu K, Jiang X, Gao M, Wang G, Liu Y, Yao Y, Chen X, Ma W, Zhang Z, Yuan Y. MYC-targeted WDR4 promotes proliferation, metastasis, and Sorafenib resistance by inducing CCNB1 translation in hepatocellular carcinoma. Cell Death Dis. 2021;12:691. 10.1038/s41419-021-03973-5. 34244479 10.1038/s41419-021-03973-5 PMC8270967

[21] Chen S, Du Y, Guan XY, Yan Q. The current status of tumor microenvironment and cancer stem cells in Sorafenib resistance of hepatocellular carcinoma. Front Oncol. 2023;13:1204513. 10.3389/fonc.2023.1204513. 37576900 10.3389/fonc.2023.1204513 PMC10412930

[22] Giraud J, Chalopin D, Blanc JF, Saleh M. Hepatocellular carcinoma immune landscape and the potential of immunotherapies. Front Immunol. 2021;12:655697. 10.3389/fimmu.2021.655697. 33815418 10.3389/fimmu.2021.655697 PMC8012774

[23] Fathi F, Saidi RF, Banafshe HR, Arbabi M, Lotfinia M, Motedayyen H. Changes in immune profile affect disease progression in hepatocellular carcinoma. Int J Immunopathol Pharmacol. 2022;36:3946320221078476. 10.1177/03946320221078476. 35226515 10.1177/03946320221078476 PMC8891922

[24] Wang H, Zhang H, Wang Y, Brown ZJ, Xia Y, Huang Z, Shen C, Hu Z, Beane J, Ansa-Addo EA, Huang H, Tian D, Tsung A. Regulatory T-cell and neutrophil extracellular trap interaction contributes to carcinogenesis in non-alcoholic steatohepatitis. J Hepatol. 2021;75:1271–83. 10.1016/j.jhep.2021.07.032. 34363921 10.1016/j.jhep.2021.07.032 PMC12888775

[25] Guerrero-Juarez CF, Goyal PK, Amber KT. Targeting Interleukin (IL)-4/IL-13 in immune checkpoint inhibitor-induced bullous pemphigoid: a cautionary note on the beneficial effect of T helper 2 immunity in melanoma and immunotherapy. Br J Dermatol. 2023;190:137–8. 10.1093/bjd/ljad324. 37703325 10.1093/bjd/ljad324 PMC10733625

[26] Gu JX, Huang K, Zhao WL, Zheng XM, Wu YQ, Yan SR, Huang YG, Hu P. NCAPD2 augments the tumorigenesis and progression of human liver cancer via the PI3K–Akt–mTOR signaling pathway. Int J Mol Med. 2024. 10.3892/ijmm.2024.5408. 10.3892/ijmm.2024.5408 PMC11315656 39092569

[27] Mai Y, Liao C, Wang S, Zhou X, Meng L, Chen C, Qin Y, Deng G. High glucose-induced NCAPD2 upregulation promotes malignant phenotypes and regulates EMT via the Wnt/beta-catenin signaling pathway in HCC. Am J Cancer Res. 2024;14:1685–711. 10.62347/HYNZ9211. 38726276 10.62347/HYNZ9211 PMC11076239

[28] Wei S, Dai M, Zhang C, Teng K, Wang F, Li H, Sun W, Feng Z, Kang T, Guan X, Xu R, Cai M, Xie D. KIF2C: a novel link between Wnt/beta-catenin and mTORC1 signaling in the pathogenesis of hepatocellular carcinoma. Protein Cell. 2021;12:788–809. 10.1007/s13238-020-00766-y. 32748349 10.1007/s13238-020-00766-y PMC8464548

[29] Li X, Huang W, Huang W, Wei T, Zhu W, Chen G, Zhang J. Kinesin family members KIF2C/4A/10/11/14/18B/20A/23 predict poor prognosis and promote cell proliferation in hepatocellular carcinoma. Am J Transl Res. 2020;12:1614–39. 32509165 PMC7270015

[30] Wang X, Sipila P, Si Z, Rosales JL, Gao X, Lee KY. CDK5RAP2 loss-of-function causes premature cell senescence via the GSK3beta/beta-catenin-WIP1 pathway. Cell Death Dis. 2021;13:9. 10.1038/s41419-021-04457-2. 34930892 10.1038/s41419-021-04457-2 PMC8688469

[31] Shen Y, Chen Y, Lin Y, Li Y, Liu P, Zhang B, Wang Y, Chan KC, Mak NK, Kahn M, Qi RZ, Yang H. CDK5RAP2 is a Wnt target gene and promotes stemness and progression of oral squamous cell carcinoma. Cell Death Dis. 2023;14:107. 10.1038/s41419-023-05652-z. 36774351 10.1038/s41419-023-05652-z PMC9922250

[32] He Y, Shao Y, Zhou Z, Li T, Gao Y, Liu X, Yuan G, Yang G, Zhang L, Li F. MORC2 regulates RBM39-mediated CDK5RAP2 alternative splicing to promote EMT and metastasis in colon cancer. Cell Death Dis. 2024;15:530. 10.1038/s41419-024-06908-y. 39048555 10.1038/s41419-024-06908-y PMC11269669

[33] Gu X, Yang H, Sheng X, Ko YA, Qiu C, Park J, Huang S, Kember R, Judy RL, Park J, Damrauer SM, Nadkarni G, Loos RJF, My VTH, Chaudhary K, Bottinger EP, Paranjpe I, Saha A, Brown C, Akilesh S, Hung AM, Palmer M, Baras A, Overton JD, Reid J, Ritchie M, Rader DJ, Susztak K. Kidney disease genetic risk variants alter lysosomal beta-mannosidase (MANBA) expression and disease severity. Sci Transl Med. 2021. 10.1126/scitranslmed.aaz1458. 10.1126/scitranslmed.aaz1458 PMC8627675 33441424

[34] Passos GR, De Oliveira MG, Ghezzi AC, Mello GC, Levi CA, D’ancona SA, Teixeira MN, Muscara CB, Grespan Bottoli L, De Vilela E, De Oliveira E, Antunes FZ, Monica. Periprostatic adipose tissue (PPAT) supernatant from obese mice releases Anticontractile substances and increases human prostate epithelial cell proliferation: the role of nitric oxide and adenosine. Front Pharmacol. 2023;14:1145860. 10.3389/fphar.2023.1145860. 37492091 10.3389/fphar.2023.1145860 PMC10364323

[35] Shi Y, Zhang W, Jia Q, Zhong X, Iyer P, Wu H, Yuan YC, Zhao Y, Zhang L, Wang L, Jia Z, Kuo YH, Sun Z. Cancer-associated SF3B1-K700E mutation controls immune responses by regulating T(reg) function via aberrant Anapc13 splicing. Sci Adv. 2024;10:eado4274. 10.1126/sciadv.ado4274. 39303038 10.1126/sciadv.ado4274 PMC11414738

[36] Jiang BJ, Zhan XL, Fu CZ, Wang HB, Cheng G, Zan LS. Identification of ANAPC13 gene polymorphisms associated with body measurement traits in Bos Taurus. Genet Mol Res. 2012;11:2862–70. 10.4238/2012.June.15.6. 22782628 10.4238/2012.June.15.6

[37] Tao M, Luo J, Gu T, Yu X, Song Z, Jun Y, Gu H, Han K, Huang X, Yu W, Sun S, Zhang Z, Liu L, Chen X, Zhang L, Luo C, Wang Q. LPCAT1 reprogramming cholesterol metabolism promotes the progression of esophageal squamous cell carcinoma. Cell Death Dis. 2021;12:845. 10.1038/s41419-021-04132-6. 34518524 10.1038/s41419-021-04132-6 PMC8438019

[38] Li Z, Hu Y, Zheng H, Li M, Liu Y, Feng R, Li X, Zhang S, Tang M, Yang M, Yu R, Xu Y, Liao X, Chen S, Qian W, Zhang Q, Tang D, Li B, Song L, Li J. LPCAT1-mediated membrane phospholipid remodelling promotes ferroptosis evasion and tumour growth. Nat Cell Biol. 2024;26:811–24. 10.1038/s41556-024-01405-y. 38671262 10.1038/s41556-024-01405-y

[39] Liu R, Yin C, Zhao P, Guo B, Ke W, Zheng X, Xie D, Wang Y, Wang G, Jia Y, Gao Y, Hu W, Liu GL, Song Z. Nuclear respiratory factor 1 drives hepatocellular carcinoma progression by activating LPCAT1-ERK1/2-CREB axis. Biol Direct. 2023;18:67. 10.1186/s13062-023-00428-z. 37875967 10.1186/s13062-023-00428-z PMC10594727

[40] He RQ, Li JD, Du XF, Dang YW, Yang LJ, Huang ZG, Liu LM, Liao LF, Yang H, Chen G. LPCAT1 overexpression promotes the progression of hepatocellular carcinoma. Cancer Cell Int. 2021;21:442. 10.1186/s12935-021-02130-4. 34419067 10.1186/s12935-021-02130-4 PMC8380368

